# Butorphanol Promotes Macrophage Phenotypic Transition to Inhibit Inflammatory Lung Injury *via* κ Receptors

**DOI:** 10.3389/fimmu.2021.692286

**Published:** 2021-07-07

**Authors:** Guangxin Luan, Fan Pan, Lina Bu, Kaixuan Wu, Aizhong Wang, Xiaotao Xu

**Affiliations:** Department of Anesthesiology, Affiliated Shanghai Sixth People’s Hospital, Shanghai Jiao Tong University, Shanghai, China

**Keywords:** butorphanol, acute lung injury, inflammation, macrophage, κ receptor

## Abstract

Acute lung injury (ALI)/acute respiratory distress syndrome (ARDS) is characterized by diffuse inflammation of the lung parenchyma and refractory hypoxemia. Butorphanol is commonly used clinically for perioperative pain relief, but whether butorphanol can regulate LPS-induced alveolar macrophage polarization is unclear. In this study, we observed that butorphanol markedly attenuated sepsis-induced lung tissue injury and mortality in mice. Moreover, butorphanol also decreased the expression of M1 phenotype markers (TNF-α, IL-6, IL-1β and iNOS) and enhanced the expression of M2 marker (CD206) in alveolar macrophages in the bronchoalveolar lavage fluid (BALF) of LPS-stimulated mice. Butorphanol administration reduced LPS-induced numbers of proinflammatory (M1) macrophages and increased numbers of anti-inflammatory (M2) macrophages in the lungs of mice. Furthermore, we found that butorphanol-mediated suppression of the LPS-induced increases in M1 phenotype marker expression (TNF-α, IL-6, IL-1β and iNOS) in bone marrow-derived macrophages (BMDMs), and this effect was reversed by κ-opioid receptor (KOR) antagonists. Moreover, butorphanol inhibited the interaction of TLR4 with MyD88 and further suppressed NF-κB and MAPKs activation. In addition, butorphanol prevented the Toll/IL-1 receptor domain-containing adaptor inducing IFN-β (TRIF)-mediated IFN signaling pathway. These effects were ameliorated by KOR antagonists. Thus, butorphanol may promote macrophage polarization from a proinflammatory to an anti-inflammatory phenotype secondary to the inhibition of NF-κB, MAPKs, and the TRIF-mediated IFN signaling pathway through κ receptors.

**Graphical Abstract:**
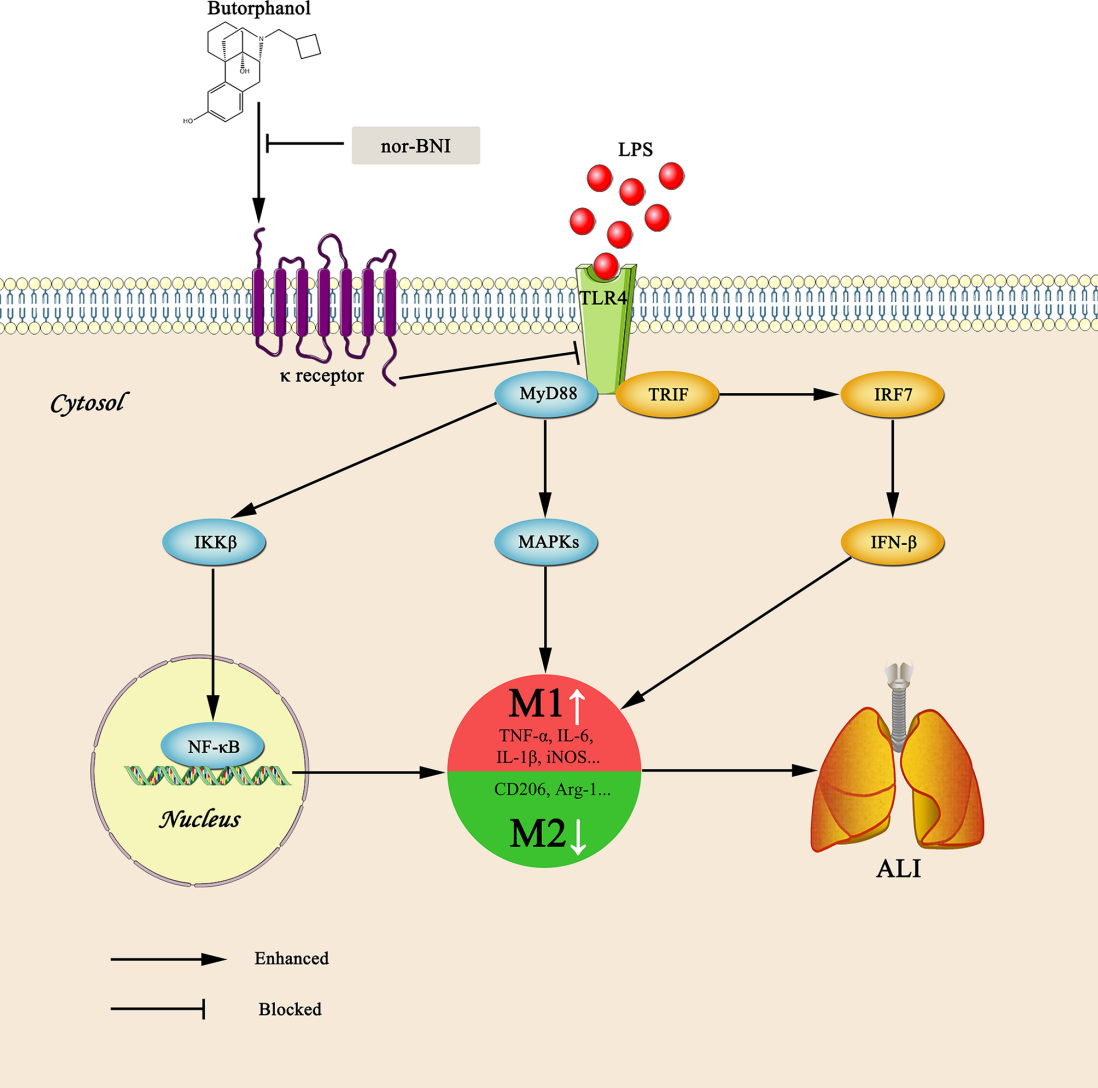
Graphical summary of the effects and mechanisms of butorphanol on sepsis-induced ALI. Upon binding of LPS to TLR4, macrophages are activated and accumulated at the site of infection where they are polarized into the M1 (a pro-inflammatory) and M2 (an anti-inflammatory) phenotypes. However, butorphanol may mitigate sepsis-induced ALI by promoting macrophage transition from M1 to M2 phenotype secondary to the inhibition of NF-κB and MAPKs, and the TRIF-mediated IFN signaling pathway through κ receptors.

## Introduction

Inflammatory responses are initiated by tissue injury or infection (1). Despite their role in clearing pathogens, uncontrolled inflammatory responses can lead to tissue structure damage (2). Sepsis is induced by potentially fatal systemic inflammatory responses secondary to infections and is the primary cause of death in intensive care units (ICUs) (3). Studies have illustrated that coagulation dysfunction, immune dysregulation and inflammatory reactions contribute to sepsis pathogenesis (4). In addition, ALI or ARDS may occur during severe sepsis (5, 6). Excessive macrophage activation has been viewed as one of the most important factors that exacerbate sepsis-induced ALI (7).

Macrophages in ALI are activated *via* Toll-like receptors (TLRs) through the recognition of damage-associated molecular patterns (DAMPs) and pathogen-associated molecular patterns (PAMPs). LPS-induced TLR4 activation in macrophages triggers the production of proinflammatory cytokines, including TNF-α, IL-6 and IL-1β, which leads to the recruitment of neutrophils and lymphocytes to the site of infection and contributes to pathogen clearance (8, 9). However, as an essential part of the innate immune system, macrophages exhibit marked plasticity and functional heterogeneity based on the cytokines to which they are exposed (10, 11). Macrophages are divided into alternatively activated (M2) macrophages and classically activated (M1) macrophages. In the early stage of inflammation, LPS can convert macrophages to an M1 phenotype, and M1 macrophages produce proinflammatory factors such as reactive oxygen intermediates and reactive nitrogen intermediates and the cytokines TNF-α, IL-6 and IL-1β (12, 13). Therefore, M1 macrophages are involved in inhibiting cellular proliferation and causing tissue injury. M2 macrophages are polarized in response to IL-10, glucocorticoids, and IL-4 (9, 11, 14) and produce anti-inflammatory cytokines and other effectors, such as IL-10, Mrc 1 (CD206), and arginase-1 (Arg-1), which take part in wound healing and tissue repair (13, 15). Although the mechanism of macrophage polarization *in vivo* is poorly understood, TLR4 induces the activation of NF-κB and further increases the induction of specific cytokines and transcription factors, which may play vital roles in controlling macrophage plasticity (9, 16–19).

Butorphanol is a lipid-soluble anesthetic drug that is a strong κ-opioid receptor (KOR) agonist and a weak μ-opioid receptor (MOR) agonist-antagonist (20). As a partial MOR and complete KOR agonist, butorphanol is widely used in the clinic as an analgesic, while the incidence of vomiting, gastrointestinal nausea and respiratory depression is less than that of pure MOR agonists (remifentanil, fentanyl, and sufentanil). In recent years, a large number of studies have confirmed the protective effect of KOR on the heart, brain, lung, and other important organs (21–24). The mechanisms may include inhibiting inflammation and oxidative stress (24). Moreover, TLR4 and opioid receptor interactions occur in the central nervous system (CNS), and effects on peripheral immune cells have also been demonstrated (25). However, whether butorphanol can regulate macrophage polarization in sepsis-induced ALI is not clear.

We examined the role of butorphanol in macrophage polarization and neutralizing sepsis-induced ALI in mice. We found that butorphanol was vital in promoting macrophage conversion and reducing the LPS-induced inflammatory response. We also demonstrated that butorphanol-induced macrophage reprogramming was crucial in the resolution of ALI. Butorphanol increased the expression of M2 markers (CD206 and Arg-1) but decreased the expression of M1 markers (TNF-α, IL-6, IL-1β and iNOS) in LPS-stimulated bone marrow-derived macrophages (BMDMs). This finding shows that butorphanol plays an important role in the alleviation of inflammatory lung injury *in vivo* and *in vitro* by promoting macrophage phenotypic transition.

## Methods and Materials

### Reagents and Antibodies

Butorphanol was obtained from Hengrui (Jiangsu, China). SB203580, PD98059, SP600125, Naloxegol, and Norbinaltorphimine dihydrochloride (nor-BNI) were obtained from MedChemExpress (Monmouth Junction, NJ, USA). LPS (Escherichia coli 055:B5) and DAPI were obtained from Sigma-Aldrich (St. Louis, MO, USA). CCK-8 kit was obtained from Dojindo (Kumamoto, Japan). TNF-α, IL-6 and IL-1β Elisa Kits were obtained from Novus (USA). TRIzol reagent, random hexamers and MultiScribe reverse transcriptase and SYBR Green PCR Master Mix were obtained from Thermo Fisher (USA). The primary antibodies include: phospho-JNK (1:1000; Cell Signaling Technology, Danvers, MA, USA), JNK (1:1000; Cell Signaling Technology, Danvers, MA, USA), phospho-ERK (1:1000; Cell Signaling Technology, Danvers, MA, USA), ERK (1:1000; Cell Signaling Technology, Danvers, MA, USA), phospho-p38 (1:1000; Cell Signaling Technology, Danvers, MA, USA), p38 (1:1000; Cell Signaling Technology, Danvers, MA, USA), phospho-STAT6 (1:1000; Cell Signaling Technology, Danvers, MA, USA), STAT6 (1:1000; Cell Signaling Technology, Danvers, MA, USA), Arg-1 (1:1000; Cell Signaling Technology, Danvers, MA, USA), F4/80 (1:1000; Cell Signaling Technology, Danvers, MA, USA), iNOS (1:1000; HUABIO, China), CD206 (1:1000; Abcam, Cambridge, UK), TLR4 (1:200; Novus, USA), MyD88 (1:200, Santa Cruz Biotechnology, CA), NF-κB (1:200; Cell Signaling Technology, Danvers, MA, USA) and β-Tubulin (1:1000; Cell Signaling Technology, Danvers, MA, USA). The secondary antibodies include: Alexa Fluor 488-conjugated anti-mouse (1:400; Abcam, Cambridge, UK), Alexa Fluor 594-conjugated anti-rabbit (1:400; Abcam, Cambridge, UK), and horseradish peroxidase (HRP)-conjugated anti-rabbit (1:10000; Cell Signaling Technology, Danvers, MA, USA).

### Animals

All studies involving animals were performed in compliance with the ARRIVE guidelines. C57BL/6J mice (males, 6-8 weeks old, 20-25 g body weight; Fudan University Medical Animal Center, Shanghai, China) were used. All mice were housed at a constant room temperature of 22-23°C with an alternating 12 h light/dark cycle and free access to water and standard food.

### Endotoxin-Induced ALI

Age- and weight-matched mice received a single intraperitoneal (i.p.) dose of LPS (10 mg·kg^-1^ body weight) (26). Butorphanol (4 mg·kg^-1^ or 8 mg·kg^-1^ body weight) was administered 30 min after LPS injection. Saline alone was similarly administered to control mice. For survival studies, mice were injected i.p. with 20 mg·kg^-1^ LPS and monitored four times daily for up to 3 d (26).

### Bone Marrow-Derived Macrophage Cultures

Bone marrow cells were collected by flushing the femur and tibia cavities of mice, plated at approximately 2×10^6^ cells/mL and incubated in Dulbecco’s modified Eagle’s medium supplemented with 10% (vol/vol) FBS, 1% (vol/vol) streptomycin/penicillin, and 10% (vol/vol) L929-conditioned media as previously described (27). Cells were used for experiments on day 7 of culture.

### Isolation of CD11b+ Alveolar Macrophages

CD11b+ alveolar macrophages were isolated according to the manufacturer’s instructions (28). Briefly, the cells were collected from total bronchoalveolar lavage fluid (BALF) and incubated with CD11b+ magnetic beads (Miltenyi Biotec). Then, the cells were applied to an MS column (Miltenyi) to select CD11b+ cells.

### CCK-8 Cell Viability Assay

A CCK-8 cell viability assay was used to measure cell viability as previously described (29). Briefly, BMDMs were treated with butorphanol (0, 1, 2, 4, and 8 μM) for 24 h. Next, 10 μl of CCK-8 solution was added to each well and incubated for 2 h. The absorbance of each well was measured at 450 nm using an automatic porous spectrophotometer (Molecular Devices, USA).

### Histological Examination

The lungs were isolated from mice in all groups, perfused and fixed with 4% paraformaldehyde, embedded, and cut into 5-μm sections. The sections were stained with hematoxylin and eosin (HE), and ten microscopic fields were used to assess the lung injury score based on a previous study (30). A semiquantitative scoring system was used to analyze lung injury, and the scoring criteria were as follows: 0: normal appearance; 1: mild interstitial hyperemia and polymorphonuclear leukocyte infiltration; 2: paravascular edema and moderate pulmonary structural damage; 3: massive cell infiltration and moderate alveolar structure destruction; and 4: massive cell infiltration and severe lung structural damage.

### Immunofluorescence Analysis

The sections were blocked with 3% goat serum (Millipore, S26-LITER) in Tris-buffered saline (TBS) containing 0.1% Triton X-100 for 1 h at room temperature. The slides were incubated with anti-F4/80, anti-iNOS or anti-CD206 overnight and then with secondary antibodies for 1 h at room temperature. The nuclei were stained with DAPI for 3 min at room temperature. BMDMs were cultured and seeded on 24-well glass coverslips. The cells were incubated with LPS with or without butorphanol for 24 h, washed three times with ice-cold phosphate‐buffered saline (PBS), fixed with paraformaldehyde for 10 min, and then blocked with 3% bovine serum albumin in PBS for 1 h at room temperature. The cells were incubated with anti-TLR4, anti-MyD88 or anti-NF-κB antibodies at 4°C overnight, washed with 1% PBST three times and incubated with secondary antibodies for 1 h at room temperature. The nuclei were stained with DAPI for 3 min at room temperature. Then, images were obtained by a Zeiss LSM 710 confocal microscope (Athens, GA, USA).

### MPO Assay

Myeloperoxidase (MPO) activity in lung tissue was measured as described previously (31). Briefly, lungs were perfused with PBS to remove all blood, weighed and homogenized in 1 mL of PBS with 0.5% hexadecyltrimethylammonium bromide. The supernatant was then collected and mixed 1/30 (vol/vol) with assay buffer (0.2 mg·mL^-1^ o-dianisidine hydrochloride and 0.0005% H_2_O_2_) after the homogenates were sonicated and centrifuged at 40,000 × g for 20 min. The change in absorbance was measured at 460 nm for 3 min, and MPO activity was calculated as the change in absorbance over time.

### Western Blotting

Protein lysates (30 µg) were resolved by 8-15% SDS-polyacrylamide gels and transferred to polyvinylidene fluoride (PVDF) membranes. The membrane was blocked with 5% dry milk in TBS containing 0.1% Tween-20 for 1 h at room temperature. The membranes were then probed with the indicated primary antibodies overnight at 4°C. Next, the membranes were washed three times and then incubated with an HRP-conjugated secondary antibody for 1 h and developed using an enhanced chemiluminescence (ECL) detection kit (Millipore, USA). Images were acquired using ImageQuant LAS 4000 mini (GE Healthcare Life Sciences, USA). Band intensity was determined by densitometric analysis with ImageJ software.

### Cytokine ELISA

Cytokines (TNF-α, IL-6 and IL-1β) in BALF were measured with a commercially available ELISA kit according to the manufacturer’s instructions.

### RNA Extraction and qRT-PCR

Total RNA was isolated from cells and reverse-transcribed with random hexamers and MultiScribe reverse transcriptase. The obtained cDNA was mixed with SYBR Green PCR Master Mix, and GAPDH served as an internal control. Relative gene expression was calculated and normalized using the 2-ΔΔCt method. The following mouse primers were used:

iNOS (forward, 5’-AGTCTCAGACATGGCTTGCCCCT-3’; reverse, 5’-GCTGCGGGGAGCCATTTTGGT-3’), IL-6 (forward, 5’-TCCAGTTGCCTTCTTGGGACTG-3’; reverse, 5’-AGCCTCCGACTTGTGAAGTGGT-3’), TNF-α (forward, 5’-GACCCTCACACTCAGATCATCT-3’; reverse, 5’-CCTCCACTTGGTGGTTTGCT-3’), IL-1β (forward, 5’-GAATCTATACCTGTCCTGTG-3’; reverse, 5’-TTATGTCCTGACCACTGTTG-3’), CD206 (forward, 5’-CCACGGATGACCTGTGCTCGAG-3’; reverse, 5’- ACACCAGAGCCATCCGTCCGA-3’), and Arg-1 (forward, 5’-GAATGGAAGAGTCAGTGTGG-3’; reverse, 5’-AATGACACATAGGTCAGGGT-3’), IRF7 (forward, 5’-GCTCCAAACCCCAAGCCCTCTG-3’; reverse, 5’-GACAGCTTCCACCTGCCATGCT-3’), IRF3 (forward, 5’-ACGGCAGGACGCACAGATGG-3’; reverse, 5’- TCCAGGTTGACACGTCCGGC-3’), IFN-β (forward, 5’-GGATCCTCCACGCTGCGTTCC-3’; reverse, 5’-CCGCCCTGTAGGTGAGGTTGA-3’).

### Lung Wet/Dry Weight Ratio

The wet/dry weight (W/D) ratios were calculated as an indicator of pulmonary edema (32). Briefly, the right upper lung of each mouse was removed, rinsed in saline and then weighed to determine the wet weight. The lung was then dried at 80°C in an oven for 48 h and reweighed to obtain the dry weight. The W/D ratios were then calculated by dividing the wet weight by the dry weight.

### Collection of BALF and Cells

BALF was collected at 24 h by cannulating a catheter into the trachea, which was then lavaged three times with 0.8 mL of cold PBS. Samples were centrifuged at 700 ×g for 5 min at 4°C. The collected supernatant was further analyzed for inflammatory cytokine levels.

### Statistical Analysis

All data are expressed as the means ± SEM. Statistical significance was determined using GraphPad Prism 8 software (GraphPad Software Inc., San Diego, CA, USA). Statistical comparisons among multiple groups were carried out using one-way ANOVA followed by Tukey’s *post hoc* test and the log-rank test. A value of P < 0.05 was considered to be statistically significant.

## Results

### Butorphanol Alleviates LPS-Induced ALI/ARDS by Regulating Macrophage Polarization *In Vivo*

**Figure 1:**
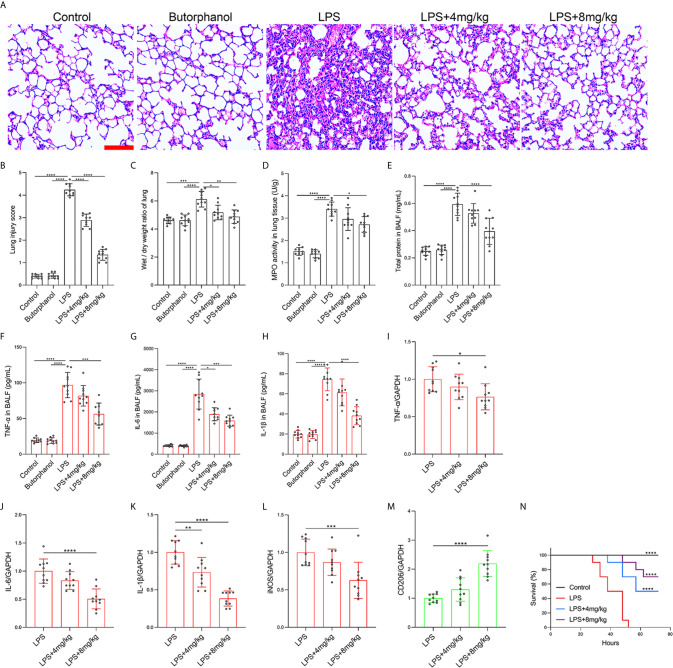
Butorphanol ameliorated LPS-induced inflammatory lung injury. Mice were injected with LPS **(**10 mg·kg^-1^ i.p.) or butorphanol (8 mg·kg^-1^ i.p.) or butorphanol (4 mg·kg^-1^ i.p. or 8 mg·kg^-1^ i.p.) plus LPS (10 mg·kg^-1^ i.p.) and analyzed after 24 h. **(A)** Hematoxylin and eosin staining of lung sections. Scale bar: 50 μm. **(B)** Lung injury score. **(C, D)** Lungs obtained at 24 h after LPS challenge were used to determine the wet/dry weight ratio and MPO activity. **(E)** The total protein level in BALF was measured with a BCA assay. **(F–H)** The BALF expression levels of TNF-α, IL-6, and IL-1β were determined by ELISA. **(I–M)** The mRNA levels of TNF-α, IL-6, IL-1β, iNOS, and CD206 in CD11b+ alveolar macrophages in the BALF of mice were measured with qRT-PCR. **(N)** Mouse survival was monitored for 72 h after a lethal dose of LPS (20 mg·kg^-1^ i.p.) alone or in combination with butorphanol (4 mg·kg^-1^ i.p. or 8 mg·kg^-1^ i.p.) 30 min after LPS challenge. n = 10 per group. The results are the means ± SEM of ten independent experiments. Statistical analysis was performed by one-way ANOVA followed by Tukey’s *post hoc* test when comparing multiple independent groups and the log-rank test. *P < 0.05, **P < 0.01, ***P < 0.001, ****P < 0.0001.

**Figure 2:**
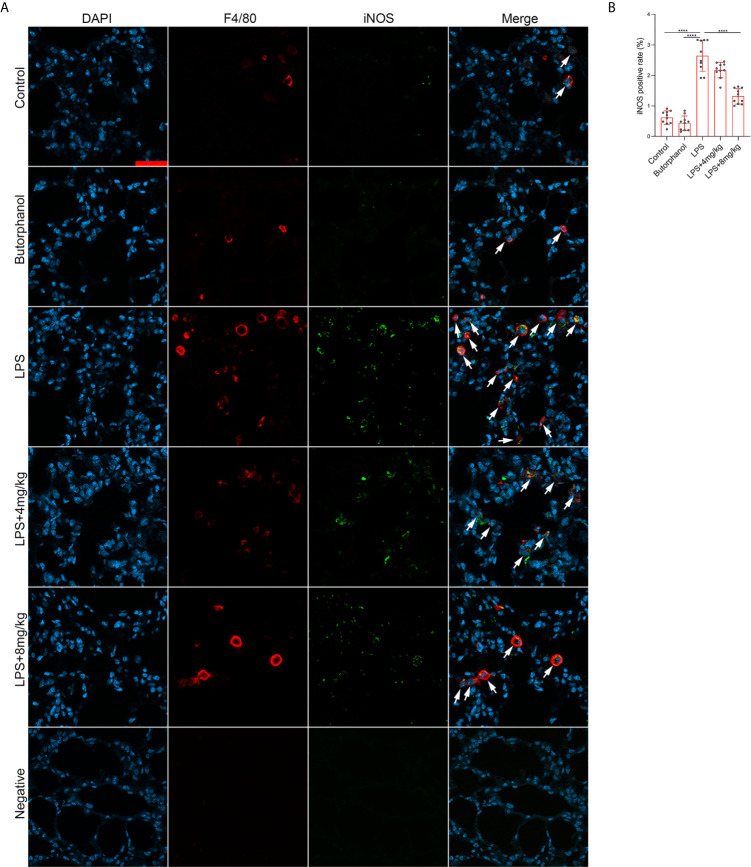
Butorphanol administration inhibited M1 macrophage polarization in LPS-induced lung injury. Mice were injected with LPS **(**10 mg·kg^-1^ i.p.) or butorphanol (8 mg·kg^-1^ i.p.) or butorphanol (4 mg·kg^-1^ i.p. or 8 mg·kg^-1^ i.p.) plus LPS (10 mg·kg^-1^ i.p.) and analyzed after 24 h. **(A)** Sections were stained with antibodies against F4/80 (red) and iNOS (green). Scale bar: 25 μm. **(B)** The image analysis results are presented as the percentage of iNOS-positive F4/80 cells. The results are the means ± SEM of ten independent experiments. Statistical analysis was performed by one-way ANOVA followed by Tukey’s *post hoc* test when comparing multiple independent groups. ****P < 0.0001.

**Figure 3:**
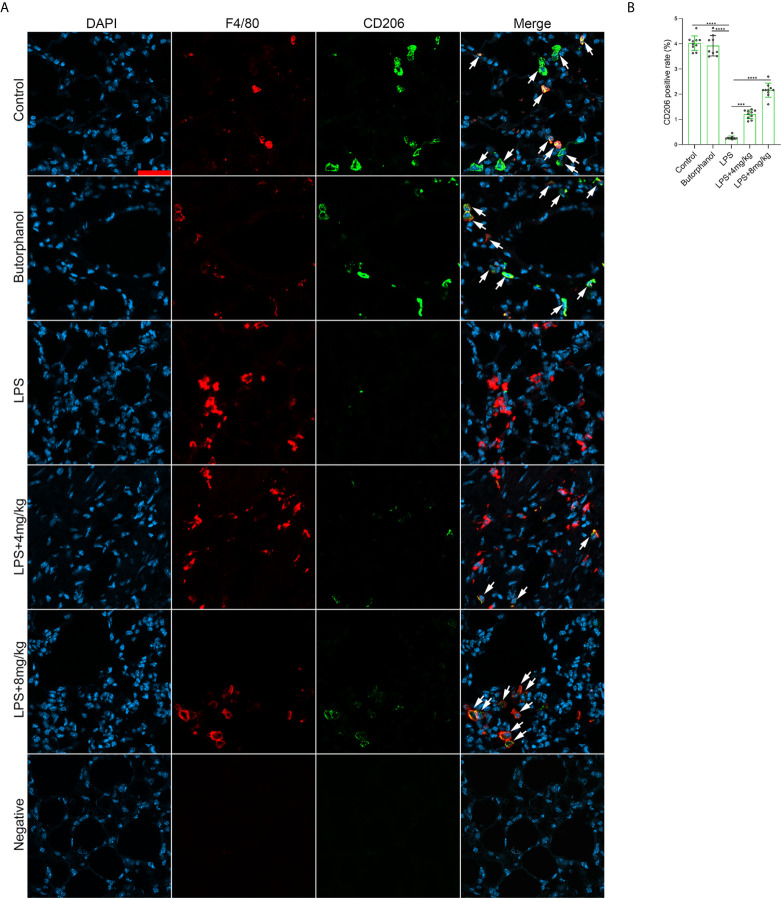
Butorphanol administration promoted M2 macrophage polarization in LPS-induced lung injury. Mice were injected with LPS **(**10 mg·kg^-1^ i.p.) or butorphanol (8 mg·kg^-1^ i.p.) or butorphanol (4 mg·kg^-1^ i.p. or 8 mg·kg^-1^ i.p.) plus LPS (10 mg·kg^-1^ i.p.) and analyzed after 24 h. **(A)** Sections were stained with antibodies against F4/80 (red) and CD206 (green). Scale bar: 25 μm. **(B)** The image analysis results are presented as the percentage of CD206-positive F4/80 cells. The results are the means ± SEM of ten independent experiments. Statistical analysis was performed by one-way ANOVA followed by Tukey’s *post hoc* test when comparing multiple independent groups. ***P < 0.001, ****P < 0.0001.

To explore the role of butorphanol in macrophage polarization and the inflammatory response *in vivo*, we established a mouse ALI/ARDS model induced by LPS (33). Histopathological analysis showed that butorphanol (4 mg·kg^-1^ or 8 mg·kg^-1^) attenuated LPS-induced lung tissue damage (**Figures 1A, B**). LPS increased the W/D ratio of lung tissues at 24 h, and this effect was reversed by butorphanol (**Figure 1C**). In addition, neutrophil sequestration in lung tissue was measured by determining MPO levels. We found that MPO activity was significantly increased in lung tissue in mice stimulated with LPS, and this effect was also neutralized by butorphanol (**Figure 1D**). In addition, butorphanol (4 mg·kg^-1^ or 8 mg·kg^-1^) significantly reversed the decrease in total protein in BALF in LPS-injected mice (**Figure 1E**). The ELISA results showed that LPS increased proinflammatory cytokine expression levels (TNF-α, IL-6, and IL-1β) in BALF. Conversely, butorphanol (4 mg·kg^-1^ or 8 mg·kg^-1^) injection decreased the LPS-induced increases in proinflammatory cytokine expression levels (TNF-α, IL-6, and IL-1β) in BALF (**Figures 1F–H**). qRT-PCR analysis showed that LPS increased the induction of proinflammatory gene (TNF-α, IL-6, IL-1β and iNOS) and reduced anti-inflammatory markers (CD206) in CD11b+ alveolar macrophages isolated from the BALF of mice. Conversely, butorphanol decreased the LPS-induced induction of proinflammatory gene (TNF-α, IL-6, IL-1β and iNOS) and enhanced anti-inflammatory markers (CD206) in cells (**Figures 1I–M**). Moreover, all mice subjected to LPS died within 48 h. The administration of butorphanol (4 mg·kg-1 or 8 mg·kg-1) resulted in 50% and 80% survival in LPS-induced mice, respectively (**Figure 1N**). We further examined the numbers of M1 and M2 macrophages in the lung by immunofluorescence. LPS increased the number of F4/80^+^iNOS^+^ M1 alveolar macrophages and decreased the number of F4/80^+^CD206^+^ M2 alveolar macrophages in lung tissues, and this effect was counteracted by butorphanol (**Figures 2A, B**; **3A, B**). These data showed that butorphanol could relieve septic ALI/ARDS by modulating macrophage polarization and inhibiting the inflammatory response *in vivo*.

### Butorphanol Inhibits M1 Macrophage Polarization

**Figure 4:**
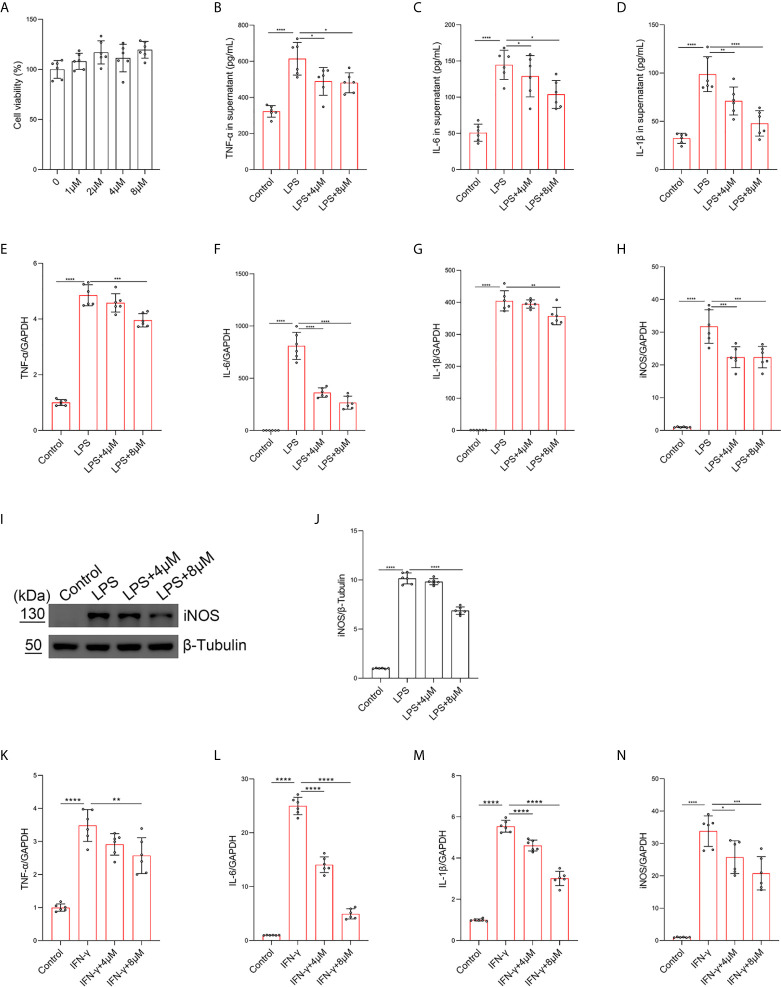
Butorphanol inhibited M1 macrophage activation. Macrophages were collected and cultured from the bone marrow of mice and treated with different concentrations of butorphanol (0-8 μM) for 24 h. **(A)** CCK-8 analysis of cell viability. Cells were incubated with LPS (1 μg·mL^-1^) alone or butorphanol (4 μM or 8 μM) plus LPS (1 μg·mL^-1^) for 24 h. **(B–D)** TNF-α, IL-6, and IL-1β protein levels in the supernatant of macrophage cultures were determined by ELISA. **(E–H)** The mRNA expression of proinflammatory cytokines (TNF-α, IL-6, IL-1β, and iNOS) in cells was measured by qRT-PCR. **(I)** The levels of iNOS were measured by western blot analysis. **(J)** Densitometric analysis of iNOS levels in **(I)** was performed with normalization to β-tubulin. Cells were incubated with IFN-γ (100 ng·mL^-1^) alone or butorphanol (4 μM or 8 μM) plus IFN-γ (100 ng·mL^-1^) for 24 h. **(K–N)** The mRNA expression of proinflammatory cytokines (TNF-α, IL-6, IL-1β, and iNOS) in cells was measured by qRT-PCR. The results are the means ± SEM of six independent experiments. Statistical analysis was performed by one-way ANOVA followed by Tukey’s *post hoc* test when comparing multiple independent groups. *P < 0.05, **P < 0.01, ***P < 0.001, ****P < 0.0001.

To further investigate the role of butorphanol in the balance of M1/M2 macrophage polarization in LPS-induced ALI, BMDMs were stimulated with LPS. The results showed that butorphanol (8 mu;Mu;) did not cause cytotoxicity in BMDMs (**Figure 4A**). We found that LPS obviously increased the expression levels of iNOS, IL-6, TNF-α and IL-1β in M1-polarized BMDMs. In contrast, 8 mu;Mu; butorphanol significantly reduced the expression levels of iNOS, IL-6, TNF-α and IL-1β in M1-polarized BMDMs stimulated with LPS (**Figures 4B–H**). The western blot results demonstrated that LPS significantly increased the level of iNOS in M1-polarized BMDMs. However, butorphanol significantly decreased the expression of iNOS in M1-polarized BMDMs stimulated with LPS (**Figures 4I, J**). This result suggests that the effect of butorphanol is TLR4-dependent. Furthermore, whether butorphanol reduced interferon (IFN)-γ-induced M1 polarization was also examined. IFN-γ significantly increased the expression levels of TNF-α, IL-6, IL-1β and iNOS in M1-polarized BMDMs. However, 8 mu;Mu; butorphanol significantly reduced the expression levels of TNF-α, IL-6, IL-1β and iNOS in M1-polarized BMDMs subjected to IFN-γ (**Figures 4K–N**). Collectively, these results showed that butorphanol alleviated the inflammatory response by inhibiting LPS- or IFN-γ-induced M1 activation of BMDMs.

### Butorphanol Promotes M2 Macrophage Polarization

**Figure 5:**
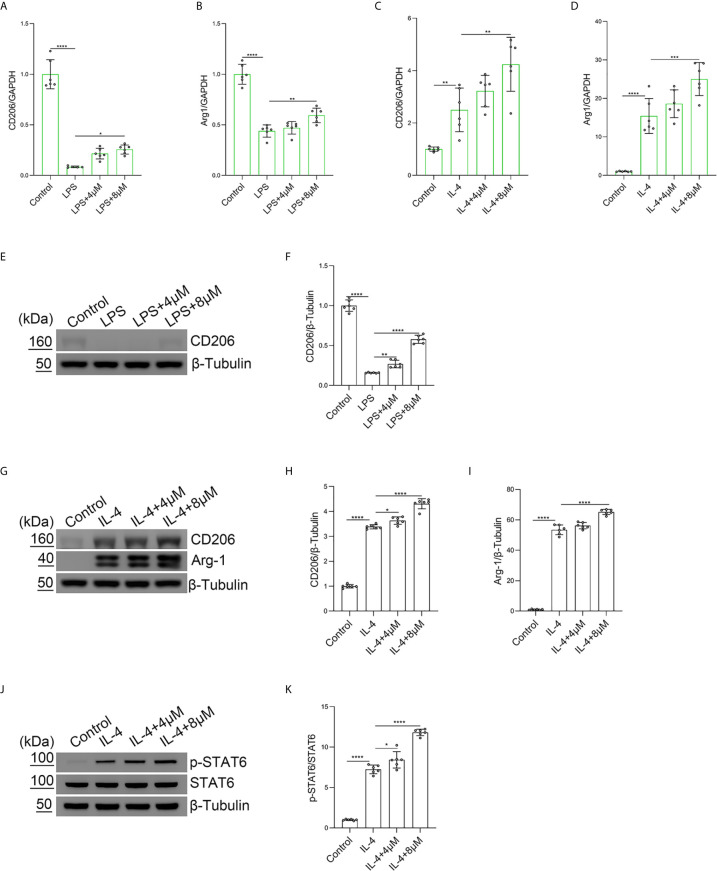
Butorphanol promoted M2 macrophage polarization. Macrophages were collected and cultured from the bone marrow of mice and incubated with LPS (1 μg·mL^-1^) alone or butorphanol (4 μM or 8 μM) plus LPS (1 μg·mL^-1^) for 24 h. **(A, B)** The mRNA expression of M2 markers (CD206 and Arg-1) in cells was measured by qRT-PCR. **(E)** The protein levels of CD206 were measured by western blot analysis. **(F)** Densitometric analysis of CD206 expression in **(E)** was performed with normalization to β-tubulin. Macrophages were incubated with IL-4 (20 ng·mL^-1^) alone or butorphanol (4 μM or 8 μM) plus IL-4 (20 ng·mL^-1^) for 24 h. **(C, D)** The mRNA expression of M2 markers (CD206 and Arg-1) in cells was measured by qRT-PCR. **(G)** The protein levels of CD206 and Arg-1 were measured by western blot analysis. **(H, I)** Densitometric analysis of CD206 and Arg-1 levels in **(G)** was performed with normalization to β-tubulin. Macrophages were incubated with IL-4 (20 ng·mL^-1^) alone or butorphanol (4 μM or 8 μM) plus IL-4 (20 ng·mL^-1^) for 30 min. **(J)** The protein levels of phosphorylated STAT6 and total STAT6 were measured by western blot analysis. **(K)** Densitometric analysis of the phosphorylated STAT6 levels in **(J)** was performed with normalization to the respective total protein. The results are the means ± SEM of six independent experiments. Statistical analysis was performed by one-way ANOVA followed by Tukey’s *post hoc* test when comparing multiple independent groups. *P < 0.05, **P < 0.01, ***P < 0.001, ****P < 0.0001.

To explore the role of butorphanol in LPS-induced M2 macrophage polarization, BMDMs were treated with LPS. The results showed that LPS obviously decreased the expression of Arg-1 and CD206 in BMDMs. In contrast, butorphanol significantly reversed the LPS-induced decrease in the expression of CD206 and Arg-1 in BMDMs (**Figures 5A, B**). Furthermore, we used IL-4 to stimulate BMDMs toward the M2 phenotype. The results showed that butorphanol increased the expression of CD206 and Arg-1 (**Figures 5C, D**). The western blot results showed that LPS significantly decreased the expression of CD206 in M1-polarized BMDMs. However, butorphanol significantly increased the expression of CD206 in M1-polarized BMDMs stimulated with LPS (**Figures 5E, F**). The western blot results also demonstrated that IL-4 significantly enhanced the expression of CD206 and Arg-1 in M2-polarized BMDMs. Moreover, butorphanol enhanced the expression of CD206 and Arg-1 (**Figures 5G–I**). It is well known that the activation of signal transducer and activator of transcription 6 (STAT6) is associated with M2 macrophage polarization (34). Therefore, the phosphorylation level of STAT6 was analyzed by western blotting. The results showed that IL-4 significantly increased the phosphorylation level of STAT6 in M2-polarized BMDMs. However, butorphanol further increased the phosphorylation level of STAT6 in M2-polarized BMDMs in the presence of IL-4 (**Figures 5J, K**). These results suggested that butorphanol was crucial in contributing to the M2 polarization of BMDMs.

### Butorphanol Modulates Macrophage Polarization Through KOR in LPS-Induced BMDMs

**Figure 6:**
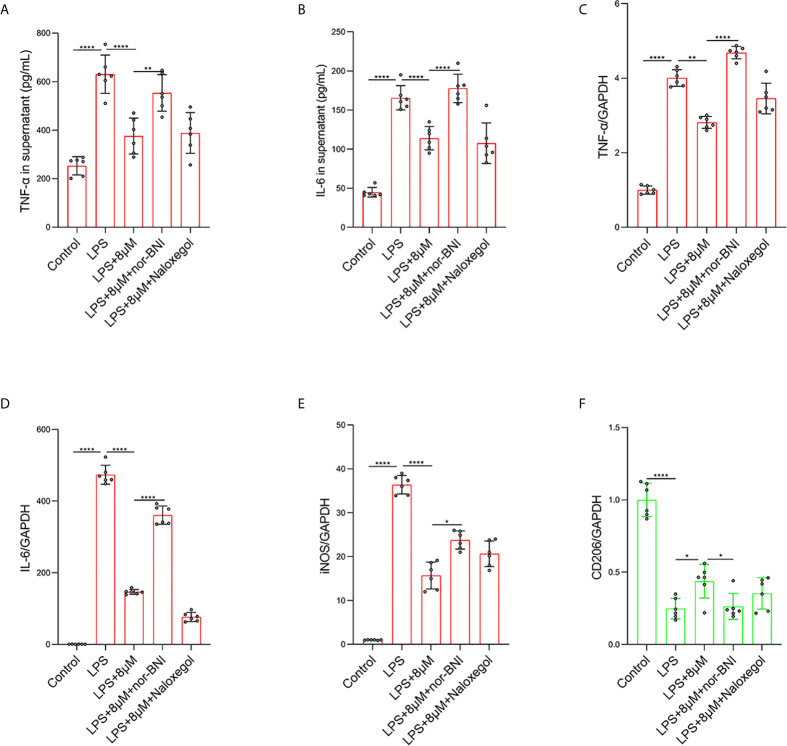
Butorphanol regulated macrophage polarization through the KOR in LPS-induced BMDMs. Macrophages were collected and cultured from the bone marrow of mice and incubated with LPS (1 μg·mL^-1^) alone or butorphanol (8 μM) plus LPS (1 μg·mL^-1^), nor-BNI (KOR antagonist, 5 μM) plus butorphanol (8 μM) plus LPS (1 μg·mL^-1^), or naloxegol (MOR antagonist, 5 μM) plus butorphanol (8 μM) plus LPS (1 μg·mL^-1^) for 24 h. Cells were pretreated with nor-BNI or naloxegol for 30 min. **(A, B)** TNF-α and IL-6 protein levels in the supernatant of macrophage cultures were determined by ELISA. **(C–F)** The mRNA levels of TNF-α, IL-6, iNOS and CD206 in cells were measured by qRT-PCR. The results are the means ± SEM of six independent experiments. Statistical analysis was performed by one-way ANOVA followed by Tukey’s *post hoc* test when comparing multiple independent groups. *P < 0.05, **P < 0.01, ****P < 0.0001.

It has been demonstrated that the activation of MOR can alleviate LPS-induced ALI through the PI3K/Akt pathway (35). To further explore which opioid receptors are involved in butorphanol-mediated macrophage polarization in LPS-induced ALI, we used MOR and KOR antagonists to block the effect of butorphanol. Compared with that in control macrophages, LPS markedly increased the expression of IL-6, TNF-α, and iNOS in BMDMs; however, butorphanol treatment notably decreased the expression of IL-6, TNF-α, and iNOS in LPS-induced BMDMs. Moreover, the inhibitory effect of butorphanol on the levels of IL-6, TNF-α, and iNOS was reversed by the KOR antagonist but not the MOR antagonist (**Figures 6A–E**). Compared with that in control macrophages, LPS markedly reduced the expression of anti-inflammatory markers (CD206) in BMDMs; however, butorphanol treatment significantly increased the expression of anti-inflammatory markers (CD206) in LPS-induced BMDMs. Moreover, the effect of butorphanol on the level of anti-inflammatory markers (CD206) was reversed by the KOR antagonist but not the MOR antagonist (**Figure 6F**).

### Butorphanol Inhibits MyD88-Dependent Signaling Pathways Through KOR in M1-Polarized BMDMs

**Figure 7:**
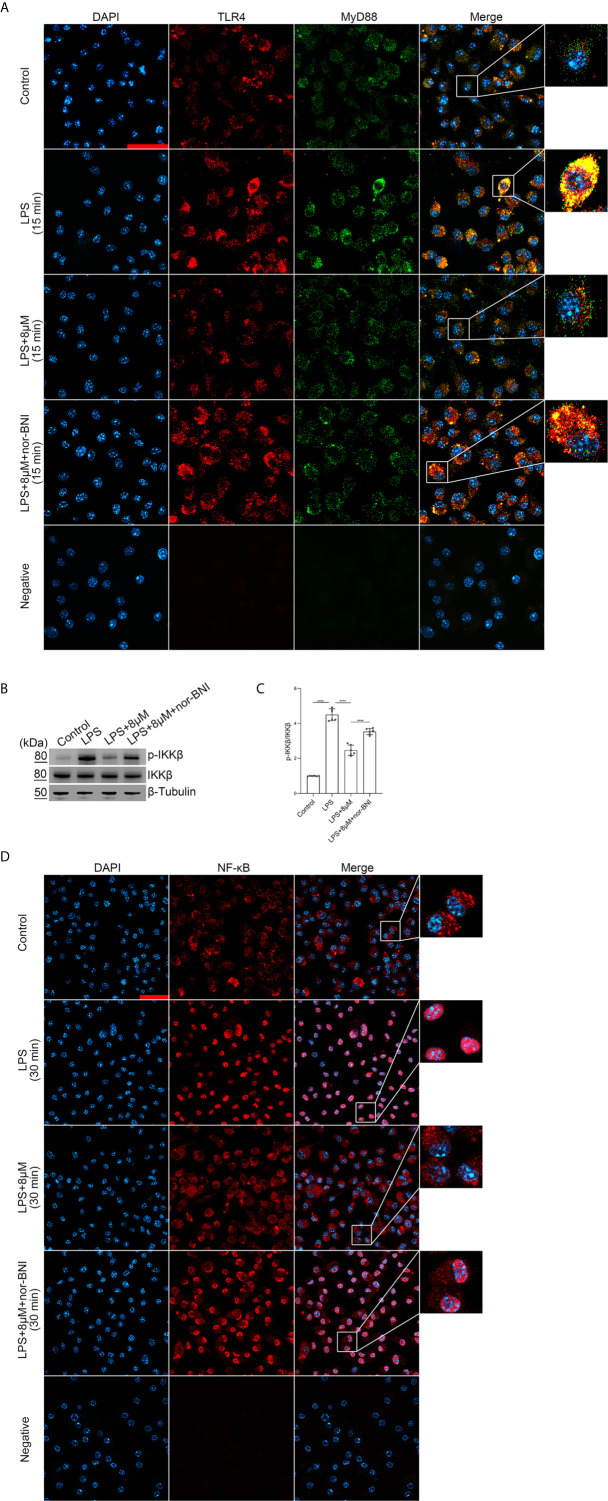
Butorphanol prevented the interaction of MyD88 with TLR4 and inhibited NF-κB activation through KOR in M1-polarized BMDMs. Macrophages were collected and cultured from the bone marrow of mice and incubated with LPS (1 μg·mL^-1^) alone, butorphanol (8 μM) plus LPS (1 μg·mL^-1^), or nor-BNI (KOR antagonist, 5 μM) plus butorphanol (8 μM) and LPS (1 μg·mL^-1^) for 15 min or 30 min. Cells were pretreated with nor-BNI for 30 min. **(A)** To observe the interaction of MyD88 with TLR4, cells were fixed, permeabilized, and stained, and images were obtained by confocal microscopy. Scale bar: 25 μm. **(B)** The levels of phosphorylated IKKβ and total IKKβ were measured by western blot analysis. **(C)** Densitometric analysis of the phosphorylated IKKβ levels in **(B)** was performed with normalization to the respective total protein. **(D)** The translocation of NF-κB in cells was examined by confocal microscopy. Scale bar: 25 μm. The results are the means ± SEM of six independent experiments. Statistical analysis was performed by one-way ANOVA followed by Tukey’s *post hoc* test when comparing multiple independent groups. ****P < 0.0001.

We explored whether butorphanol prevented TLR4 signaling, which induces the nuclear translocation of NF-κB *via* MyD88-independent and MyD88-dependent pathways (36). In our study, LPS induced a strong TLR4-MyD88 association within 15 min and IκB kinase β (IKKβ) phosphorylation and NF-κB translocation within 30 min. However, butorphanol inhibited the association of MyD88 with TLR4, IKKβ phosphorylation and NF-κB translocation (**Figures 7A–D**); thus, butorphanol prevents MyD88-dependent NF-κB activation. The role of butorphanol in preventing the association of TLR4 with MyD88, IKKβ phosphorylation and NF-κB activation was alleviated by a KOR antagonist (**Figures 7A–D**).

### Butorphanol Inhibits M1 Macrophage Polarization Through the MAPK Signaling Pathway in M1-Polarized BMDMs

**Figure 8:**
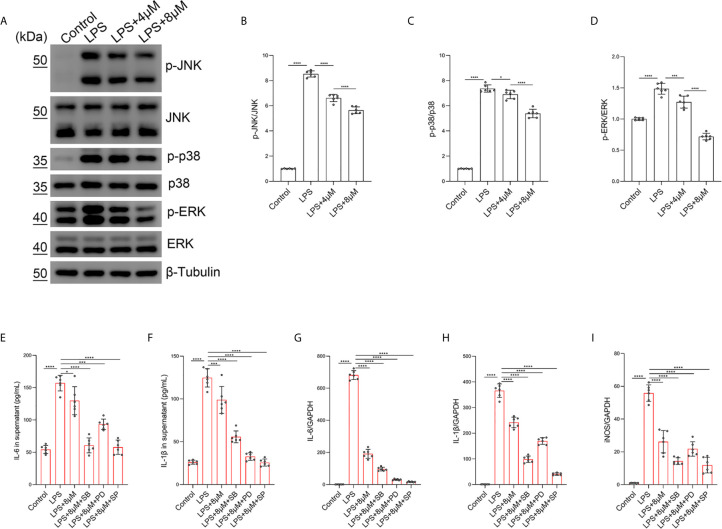
Butorphanol inhibited M1 phenotype macrophage polarization through the MAPK signaling pathway. Macrophages were collected and cultured from the bone marrow of mice and treated with LPS (1 μg·mL^-1^) alone or butorphanol (4 μM or 8 μM) plus LPS (1 μg·mL^-1^) for 30 min. **(A)** The levels of phosphorylated MAPKs and their respective total proteins were measured by western blot analysis. **(B–D)** Densitometric analysis of the phosphorylated MAPKs levels in **(A)** was performed with normalization to the respective total proteins. Macrophages were incubated with LPS (1 μg·mL^-1^) alone, butorphanol (8 μM) plus LPS (1 μg·mL^-1^), the p38 inhibitor SB203580 (10 µM) plus LPS (1 μg·mL^-1^), the ERK inhibitor PD98059 (10 µM) plus LPS (1 μg·mL^-1^), or the JNK inhibitor SP600125 (10 µM) plus LPS (1 μg·mL^-1^) for 24 h. **(E, F)** IL-6 and IL-1β protein levels in the supernatant of macrophage cultures were determined by ELISA. **(G–I)** The mRNA levels of IL-6, IL-1β, and iNOS in cells were measured by qRT-PCR. The results are the means ± SEM of six independent experiments. Statistical analysis was performed by one-way ANOVA followed by Tukey’s *post hoc* test when comparing multiple independent groups. *P < 0.05, ***P < 0.001, ****P < 0.0001.

To confirm that the JNK/ERK/P38 pathway was involved in the effect of butorphanol on M1 macrophage polarization, we examined the effect of butorphanol on LPS-induced JNK, ERK and P38 phosphorylation in BMDMs. Compared with those of control macrophages, LPS increased the phosphorylation levels of JNK, P38 and ERK within 30 min. However, LPS-induced JNK, P38 and ERK phosphorylation was decreased by butorphanol treatment (**Figures 8A–D**). Furthermore, we used the JNK inhibitor SP600125 (10 µM), the ERK inhibitor PD98059 (10 µM) and the P38 inhibitor SB203580 (10 µM) to block the phosphorylation of JNK, ERK and P38, respectively (37). As shown in **Figures 8E–I**, the JNK, ERK and P38 inhibitors significantly inhibited the expression of IL-1β, IL-6, and iNOS in LPS-treated BMDMs. Moreover, the levels of inflammatory cytokines (IL-1β, IL-6, and iNOS) in the JNK, ERK and P38 inhibitor groups were similar to those in the butorphanol treatment group. Taken together, our results demonstrated that butorphanol inhibited M1 macrophage polarization through the JNK/ERK/P38 signaling pathway in M1-polarized BMDMs.

### Butorphanol Prevents the Phosphorylation of MAPKs in M1-Polarized BMDMs Through KOR

**Figure 9:**
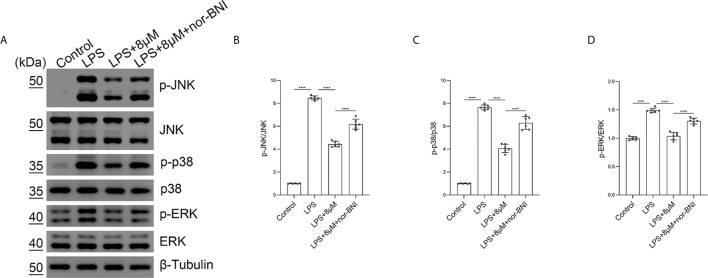
Butorphanol prevented the phosphorylation of MAPKs through KOR in M1-polarized BMDMs. Macrophages were collected and cultured from the bone marrow of mice and incubated with LPS (1 μg·mL^-1^) alone, butorphanol (8 μM) plus LPS (1 μg·mL^-1^) or nor-BNI (KOR antagonist, 5 μM) plus butorphanol (8 μM) plus LPS (1 μg·mL^-1^) for 30 min. Cells were pretreated with nor-BNI for 30 min. **(A)** The levels of phosphorylated MAPKs and their respective total proteins were measured by western blot analysis. **(B–D)** Densitometric analysis of the phosphorylated MAPKs levels in **(A)** was performed with normalization to the respective total proteins. The results are the means ± SEM of six independent experiments. Statistical analysis was performed by one-way ANOVA followed by Tukey’s *post hoc* test when comparing multiple independent groups. ****P < 0.0001.

Moreover, we found that butorphanol (8 mu;Mu;) administration significantly inhibited the phosphorylation of MAPKs in LPS-induced BMDMs. The effect of butorphanol on inhibiting the phosphorylation of MAPKs in LPS-induced BMDMs was counteracted by pretreatment with a KOR antagonist (**Figures 9A–D**). Taken together, our results demonstrated that butorphanol inhibited the phosphorylation of MAPKs through KOR in M1-polarized BMDMs.

### Butorphanol Inhibits TRIF-Dependent Signaling Pathways Through KOR in M1-Polarized BMDMs

**Figure 10:**
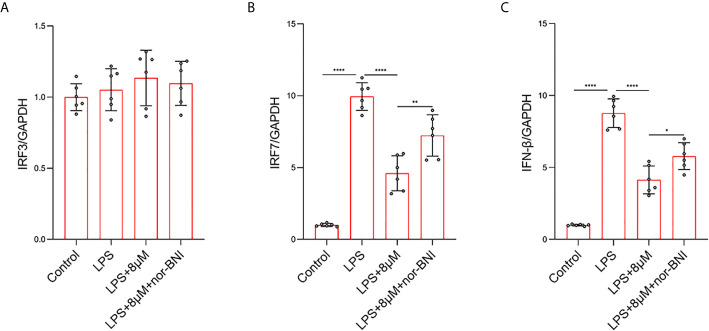
Butorphanol decreased LPS-induced the elevation of IRF7 and IFN-β through KOR in M1-polarized BMDMs. Macrophages were collected and cultured from the bone marrow of mice and incubated with LPS (1 μg·mL^-1^) alone, butorphanol (8 μM) plus LPS (1 μg·mL^-1^) or nor-BNI (KOR antagonist, 5 μM) plus butorphanol (8 μM) plus LPS (1 μg·mL^-1^) for 6 h. Cells were pretreated with nor-BNI for 30 min. **(A–C)** The mRNA levels of IRF3, IRF7, and IFN-β in cells were measured by qRT-PCR. The results are the means ± SEM of six independent experiments. Statistical analysis was performed by one-way ANOVA followed by Tukey’s *post hoc* test when comparing multiple independent groups. *P < 0.05, **P < 0.01, ****P < 0.0001.

In addition, TLR4 mediates the expression of inflammatory genes through TRIF-dependent signaling pathways in LPS-treated macrophages (38). In our study, the mRNA level of IFN-regulated factor-3 (IRF3) was not significantly increased at 6 h, whereas LPS significantly increased the elevation of IRF7 at 6 h, which are involved in the TLR4-mediated TRIF-dependent IFN signaling pathway in LPS-induced macrophages (38). Butorphanol decreased the LPS-induced elevation of IRF7 in BMDMs. The effect of butorphanol on inhibiting elevation of IRF7 was alleviated by a KOR antagonist (**Figures 10A, B**). Moreover, LPS significantly increased the mRNA level of IFN-β in BMDMs. Butorphanol significantly reduced the mRNA level of IFN-β in LPS-induced BMDMs, the effect of which was attenuated by the KOR antagonist (**Figure 10C**). Thus, butorphanol functions by inhibiting TRIF-dependent IFN signaling pathways through KOR in M1-polarized BMDMs.

## Discussion

The present study was performed to verify the hypothesis that butorphanol contributes to M2 macrophage polarization to alleviate sepsis-induced ALI and explore the potential mechanisms. Our results demonstrated that butorphanol ameliorated pathological lung damage, decreased the inflammatory response, and promoted M2 macrophage polarization in LPS-induced ALI.

Furthermore, we showed that butorphanol suppressed M1 polarization in LPS-induced BMDMs and reduced the expression of proinflammatory cytokines (IL-1β, IL-6, and TNF-α). Moreover, butorphanol increased M2 macrophage polarization by upregulating the expression of CD206 and Arg-1 in BMDMs induced by LPS or IL-4. Furthermore, butorphanol inhibited NF-κB and MAPK activation and the TRIF-mediated IFN signaling pathway through KOR in LPS-induced M1-polarized BMDMs.

Accumulating evidence has suggested that macrophage polarization plays an important role in the progression of inflammatory diseases, such as sepsis and ALI (39, 40). M1 macrophages are characterized as toxic, while M2 macrophages are considered to be protective (18). It has been demonstrated that butorphanol also has therapeutic effects on ventilator-associated lung injury (VALI) (24). The main pathological change in VALI is the destruction of pulmonary vascular endothelial cells and the increase in pulmonary vascular permeability, which is particularly significant in obese patients (41). It was suggested that butorphanol could reduce lung injury and improve intraoperative oxygenation in obese patients by inhibiting inflammation and reducing vascular injury (24). Moreover, it has been shown that butorphanol can relieve sepsis-induced brain injury by inhibiting the NF-κB signaling pathway (42). However, we further demonstrated that butorphanol promoted macrophage phenotypic transition to alleviate sepsis-induced lung injury secondary to the inhibition of NF-κB and MAPK signaling, as well as the TRIF-mediated IFN signaling pathway through KOR.

To estimate the bioactivity of butorphanol *in vivo*, we established a mouse model of LPS-induced ALI (43). The results showed that butorphanol notably increased the survival rates of mice subjected to LPS and decreased the levels of lung inflammatory cytokines. It has been noted that the lungs are susceptible to infection during sepsis (44). Therefore, preventing lung infection may be an effective therapeutic approach in sepsis. Our results demonstrated that butorphanol modified lung tissue injury, inhibited M1 macrophage polarization, and enhanced M2 macrophage polarization in the lungs of LPS-injected mice. In addition, butorphanol administration significantly enhanced the number of M2 macrophages and decreased the number of M1 macrophages in the lungs of LPS-injected mice.

The present study also verified that butorphanol significantly converted macrophages to the M2 phenotype. The features of M1 or M2 macrophage polarization include the expression of M1- or M2-associated molecular markers, respectively. For instance, M1 macrophage markers are iNOS, pSTAT1, pSTAT3, TNF-α, IL-6, IFNγ, IL-12p70, and IL-1β, whereas M2 macrophage markers are Arg-1, Chi3l3, pSTAT6, IL-4, and IL-10 (45). Butorphanol inhibited LPS-induced M1-associated molecular markers and enhanced M2-associated molecular markers in BMDMs. These results showed that butorphanol could promote M2 macrophage polarization both *in vivo* and *in vitro.* Furthermore, butorphanol treatment markedly reduced the expression of IL-6, TNF-α, and iNOS in LPS-induced BMDMs. Moreover, the inhibitory effect of butorphanol on the levels of IL-6, TNF-α, and iNOS was reversed by a KOR antagonist but not an MOR antagonist.

Potential interactions can occur between TLR4 and opioid receptors (μ, δ and κ). These interactions are involved in immune function, opioid analgesia, and intestinal motility (25). The TLR4 signaling pathway was directly activated by opioid receptor agonists in the CNS in the absence of LPS, indicating interactions within the cell membrane. Opioids bind to and activate TLR4, which in turn increases the production of proinflammatory cytokines in the CNS (46). However, opioid receptor agonists inhibit LPS-induced TLR4 signaling and decrease the inflammatory response in peripheral immune cells, indicating that the TLR4 and opioid receptor interaction is dependent on the cell type and activator (47, 48). In addition, NF-κB activation plays a central role in driving inflammatory signaling (49). Under normal conditions, NF-κB is inhibited by IκB and maintained in an inactive state in the cytoplasm (49). IKK-β induces IκBα phosphorylation (at Ser32), which causes its dissociation and degradation, promoting NF-κB release and subsequent reactive oxygen species (ROS) and cytokine production (50, 51). The formation of inactive dimers of the p50 subunit of NF-κB is essential for promoting macrophage polarization to the anti-inflammatory phenotype, resulting in the suppression of NF-κB-induced macrophage polarization (17). We demonstrated that butorphanol prevented the association of TLR4 with MyD88 and inhibited the phosphorylation of IKK-β, which restrained NF-κB signaling activation (49) and further promoted macrophages to shift to the anti-inflammatory M2 phenotype. These results demonstrated that the inhibitory effect of butorphanol on the interaction between TLR4 and MyD88, the phosphorylation of IKK-β and the activation of NF-κB were reversed by the KOR antagonist.

In addition, the MAPK pathway is downstream of the opioid receptor and TLR4 signaling pathways, and opioid receptors can also induce neuroinflammation in the CNS through the activation of MAPK signals (25). Different opioid receptors have distinct roles in different cell types and diseases. MAPK phosphorylation in murine macrophages promoted M1 macrophage polarization, resulting in cytotoxic and inflammatory effects such as NO production. MAPKs have been viewed as potential targets of sepsis. Briefly, LPS binds to TLR4 and induces the activation of MAPK-dependent intracellular signaling (41, 43, 44). In our study, the phosphorylation of JNK, ERK, and p38 was significantly increased after LPS stimulation, but butorphanol decreased the phosphorylation levels of JNK, ERK, and p38. In our study, inhibition of the phosphorylation levels of MAPKs completely blocked LPS-induced M1 macrophage polarization, demonstrating that the suppression of M1 polarization by butorphanol to exert anti-inflammatory effects may inhibit the JNK, ERK, and p38 pathways. In addition, we found that butorphanol-mediated inhibition of MAPK phosphorylation in LPS-induced BMDMs was counteracted by pretreatment with a KOR antagonist. Moreover, we demonstrated that butorphanol inhibited LPS-induced the elevation of IRF7 and IFN-β, which was attenuated by a KOR antagonist.

In summary, our study demonstrates that butorphanol alleviates LPS-induced ALI by contributing to M2 macrophage polarization and inhibiting M1 macrophage polarization. Moreover, butorphanol significantly decreases M1 polarization and increases M2 polarization in macrophages secondary to inhibition of the NF-κB and MAPKs and the TRIF-mediated IFN signaling pathway through KOR. Our results demonstrate that butorphanol may be an anti-inflammatory agent for the treatment of sepsis and ALI.

## Data Availability Statement

The original contributions presented in the study are included in the article/supplementary material. Further inquiries can be directed to the corresponding authors.

## Ethics Statement

All the procedures and all animal experiments carried out in this study were performed in accordance with the Guide for the Care and Use of Laboratory Animals (eighth edition) published by the National Research Council (United States) and were approved by the Institutional Animal Care and Use Committee of Sixth People’s Hospital affiliated with Shanghai Jiao Tong University.

## Author Contributions

GL and XX designed and performed most of the experiments, analyzed and interpreted the data, and wrote the manuscript. FP, LB and KW assisted during the acquisition, analysis, and interpretation of data and revised the manuscript. XX and AW assisted with data acquisition and manuscript revision. XX is responsible for the integrity of the work as a whole. All authors contributed to the article and approved the submitted version.

## Funding

This work was supported by the National Natural Science Foundation of China (81772062).

## Conflict of Interest

The authors declare that the research was conducted in the absence of any commercial or financial relationships that could be construed as a potential conflict of interest.

## Abbreviations

ALI, Acute lung injury; ARDS, Acute respiratory distress syndrome; VALI, Ventilator-associated lung injury; TLR4, Toll-like receptor 4; TRIF, Toll/IL-1 receptor domain-containing adaptor inducing IFN-β; PAMPs, Pathogen-associated molecular patterns; DAMPs, Damage-associated molecular patterns; LPS, Lipopolysaccharide; MPO, Myeloperoxidase; BALF, Bronchoalveolar lavage fluid; KOR, κ-opioid receptor; MOR, μ-opioid receptor; CNS, Central nervous system.
